# Newborns’ early attuning to hand‐to‐mouth coordinated actions

**DOI:** 10.1111/desc.13162

**Published:** 2021-08-03

**Authors:** Margaret Addabbo, Elisa Roberti, Lorenzo Colombo, Odoardo Picciolini, Chiara Turati

**Affiliations:** ^1^ Department of Psychology University of Milan‐Bicocca Milano Italy; ^2^ Neonatal Intensive Care Unit Fondazione IRCCS Cà Granda Ospedale Maggiore Policlinico Milan Italy; ^3^ Pediatric Physical Medicine & Rehabilitation Unit Fondazione IRCCS Ca' Granda Ospedale Maggiore Policlinico Milan Italy

**Keywords:** goal‐directed actions, hand‐to‐mouth coordination, newborns, sensorimotor experience, visual preference

## Abstract

Already inside the womb, fetuses frequently bring their hands to the mouth, anticipating hand‐to‐mouth contact by opening the mouth. Here, we explored whether 2‐day‐old newborns discriminate between hand actions directed towards different targets of the face—that is, a thumb that reaches the mouth and a thumb that reaches the chin. Newborns looked longer towards the thumb‐to‐mouth compared to the thumb‐to‐chin action only in the presence, and not absence, of anticipatory mouth opening movements, preceding the thumb arrival. Overall, our results show that newborns are sensitive to hand‐to‐face coordinated actions, being capable to discriminate between body‐related actions directed towards different targets of the face, but only when a salient visual cue that anticipates the target of the action is present. The role of newborns’ sensorimotor experience with hand‐to‐mouth gestures in driving this capacity is discussed.

## 1 INTRODUCTION

Actions intrinsically characterize human lives from very early in development. Already within the confines of the womb, fetuses perform actions directed towards the uterine environment and their bodies, especially the face (Myowa‐Yamakoshi & Takeshita, 2006; Reissland et al., 2014; Sparling et al., 1999; Zoia et al., 2007). When fetuses touch their face, they modify the kinematic of their actions relying on the specific facial region that is going to be touched (Zoia et al., 2007). Further, from 24 to 36 weeks of gestation, fetuses show increasing hand‐to‐face contact towards the lower parts of the face and the mouth area (Reissland et al., 2014). Also, the proportion of mouth movements preceding hand‐to‐mouth contacts increases with gestational age, showing that the fetuses behaviorally anticipate hand‐to‐mouth contacts by opening their mouths (Myowa‐Yamakoshi & Takeshita, 2006; Reissland et al., 2014). Thus, in the third trimester, fetal hand‐to‐mouth coordination develops from purposeless movements to organized actions, which are considered precursors of feeding behaviors after birth (Reissland et al., 2014). These body‐related exploratory actions remain prominent behaviors also in the first hours after birth and during the first months of life (DiMercurio et al., 2018; Rochat, 1993). These challenging findings show that, already at birth, newborns are active explorers of their bodies and that their actions are not purely reflexes or passive movements but, rather, organized and coordinated goal‐directed behaviors.

The aforementioned evidence prompted a handful of studies to investigate whether early motor experience could impact newborns' visual sensitivity to actions and gestures at birth. Indeed, findings have shown that newborns are already attuned to several features that characterize human actions and gestures (Craighero et al., 2020; Craighero et al., 2011; Craighero et al., 2016; Longhi et al., 2015). For example, newborns can discriminate between biomechanically possible and impossible whole hand grasping movements, showing a visual preference for the latter (Longhi et al., 2015). Further, newborns seem to be sensitive to action translational and kinematic properties, as they prefer to look at point‐light‐displays configurations of a hand performing a grasp compared to a closed hand shape when the action is directed towards the external world (Craighero et al., 2016). Also, newborn infants differentiate between constant motion and the accelerated‐decelerated kinematics that characterize biological goal‐directed motion (Craighero et al., 2020). Only one study explored newborns’ processing of goal‐directed actions, showing that 2‐day‐olds prefer to look at grasping hand actions compared to non‐grasping actions when the movement was directed away from the body (and not towards the body) and when the object to‐be‐grasped was present in the visual scene (Craighero et al., 2011). Such visual preference was interpreted by the authors as an early attuning to purposeful actions directed towards objects in the external world (Craighero et al., 2011). Overall, previous studies focused their attention on hand grasping actions directed away from the body and towards objects in the external environment.

So far, no study has investigated whether newborns are visually attuned to coordinated hand‐to‐face actions directed towards different targets of the face. Exploring hand‐to‐face actions is important for several reasons. First of all, converging evidence show that these actions are the very first forms of planned and organized behaviors (Myowa‐Yamakoshi & Takeshita, 2006; Reissland et al., 2014; Zoia et al., 2007) and are considered precursors of highly adaptive abilities that emerge later in development (i.e., soothing and feeding behaviors) (Feldman & Brody, 1978; Lew & Butterworth, 1995; Radzyminski, 2005). Second, during hand‐to‐face contacts, the body is both the agent and the target of the action, and actions directed towards the face can bypass vision and rely solely on sensorimotor and proprioceptive information.

Here, through a preferential looking paradigm and a between‐subject design, we explored whether 2‐day‐old newborns could manifest a visual preference and discriminate between actions directed towards different targets of the face—that is, a thumb that reaches the mouth and a thumb that reaches the chin—manipulating the presence of mouth opening movements preceding the thumb arrival. We hypothesized that the newborn might rely on the mouth opening that precedes the hand action to detect the final target of the action, preferring the action in which the mouth opening movement was followed by a thumb‐to‐mouth contact. Conversely, when the mouth remains closed, newborns cannot rely on a clear visual cue that helps them to detect the final target of the action; therefore, they should not manifest a visual preference for any of the two action scenes. To summarize, we expected newborns to differentiate between the two actions (thumb‐to‐month vs. thumb‐to‐chin) only in the presence of the mouth opening. The mouth‐closed condition also controlled for the possible role of perceptual features (i.e., different trajectories, kinematics, and target on the face) in driving newborns’ attention towards one of the two hand‐to‐face actions.

**RESEARCH HIGHLIGHTS**

- We explored whether newborns are visually attuned to coordinated hand‐to‐face actions directed towards different targets of the face (mouth vs. chin).
- Newborns look longer to a thumb‐to‐mouth compared to a thumb‐to‐chin action only if the mouth opens in anticipation of the thumb arrival.
- Visual preferences for hand‐to‐mouth coordinated actions might arise from early pre‐ and postnatal sensorimotor experiences with hand‐to‐face actions.

## 2 MATERIALS AND METHODS

### 2.1 Participants

Thirty‐six healthy full‐term Caucasian newborns (26 girls; mean age: 43.56 h, range: 20–77, mean birth weight: 3300 g) recruited at the maternal unit of the Ospedale Maggiore Policlinico in Milan were tested as soon as they woke up, before feeding and when they were in an awake and alert state. Half of the newborns were assigned to the mouth‐opening condition and the other half to the mouth‐closed condition. All newborns were full‐term (natural childbirth between 37 and 41 gestational age), with a normal APGAR (range 8−10), normal birth weight (range 3000−3940 g). Independent *t*‐tests showed that there were no differences between groups (mouth‐opening, mouth‐closed) in weight (*t (34)* = 1.382, *p* = 0.18), time after birth (in hours) (*t (34)* = 1.229, *p* = 0.23), and gestational age (*t (34)* = 0.827, *p* = 0.41). We have tested additional 12 newborns, but they were then excluded from the final sample due to fussiness or not being cooperative (*n* = 4 in the mouth‐opening group; *n* = 3 in the mouth‐closed group) or to a position bias (i.e., looking towards the right or the left position for more than the 85% of the total looking time) (*n* = 2 in the mouth‐opening group; *n* = 3 in the mouth‐closed group). Power analyses using effect sizes based on a previous preferential looking study with newborns (*f *= 0.30; Longhi et al., 2015) revealed that a total sample size of at least 18 participants per group would have provided enough power (0.80 with an alpha level of 0.05) to identify similar effects. Parental written informed consent was obtained before testing began. The protocol was carried out in accordance with the ethical standards of the Declaration of Helsinki (BMJ 1991; 302: 1194) and approved by the Ethics Committee of the Ospedale Maggiore Policlinico (NeoPerFiSo, n. 624/2018).

### 2.2 Stimuli

**FIGURE 1:**
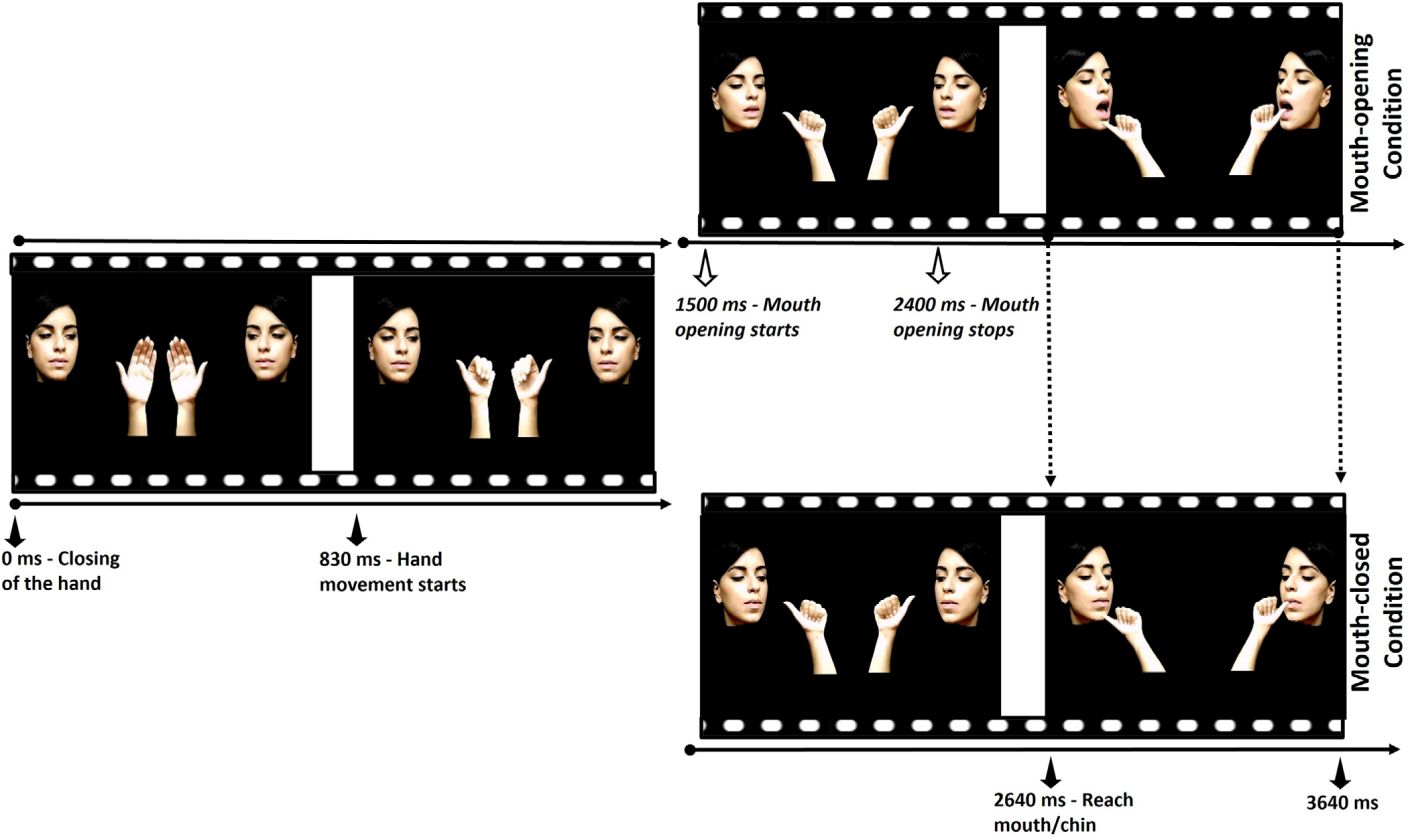
Four frames extracted from the videos showing the time‐line of the thumb‐to‐mouth versus thumb‐to‐chin actions in the mouth opening (upper panel) and mouth‐closed (lower panel) condition

Newborns were randomly assigned to one of two experimental conditions: the mouth opening or mouth‐closed condition. Stimuli in the mouth‐opening (see supplementary file S1) and mouth‐closed conditions (see supplementary file S2) were kept equal for action kinematics, trajectory, and target (mouth vs. chin). In both conditions, newborns were presented simultaneously and bilaterally on the screen with two videos depicting hand‐actions directed towards two different targets of a face. One video displayed a movement of the thumb directed towards the mouth (thumb‐to‐mouth) while, in the other video, the movement of the thumb was directed towards the chin (thumb‐to‐chin). The face targeted by the action remained constant. Each video lasted 3640 ms. The hands were initially presented centrally on the screen with the palm facing the observer. During the first 830 ms of the videos, the hands were closed, leaving the thumb out. Then, the hands moved towards the face, displayed in a more peripheral location, and reached the final target position (mouth/chin) at 2640 s from action onset, remaining still for the last 1000 ms. The head of the actress first faced the observer gazing at the hand, then begun to rotate towards the hand at 830 ms from action onset and stopped the rotation at 2000 ms. In the mouth‐opened condition, during the rotation, the actress started to open her mouth at 1500 ms from video onset and reached the maximum opening after 2400 ms (Figure 1). Stimuli were also equalized for luminance, which did not differ between the touch‐to‐chin (*M* = 14.02, *SD* = 0.31) and touch‐to‐mouth (*M* = 14.03, *SD* = 0.28) stimuli (Mann–Whitney *U* test, *p* = 0.75). In the mouth‐closed condition, the mouth remained closed during the rotation. The face and its rotation were the same in the two experimental conditions. To create the mouth‐closed condition, the mouth‐opening stimuli were modified frame‐by‐frame in Adobe Photoshop to convert the opening mouth into a closed one while keeping all the other events equal across conditions. Global luminance, hue, as well as saturation were kept constant between stimuli. Only in the last portion of the mouth‐opening video (from 2640 to 3640 ms), in which the thumb entered the opening mouth, the local contrast of the sole mouth region was slightly different from that of the thumb touching the chin because of the presence of the thumb in the mouth, which had a darker background, generating greater contrast. The difference between the final positions of the thumbs on the face (mouth vs. chin) was 4.8° (2.5 cm). In turn, the spatial frequency of this difference was 0.1 c/d at the viewing distance of 30 cm, which corresponds to the peak of sensitivity to the contrast of newborns (Atkinson, 2002; Slater & Sykes, 1977). The videos were presented bilaterally and played continuously in a loop. The dimension of the hand/arm at a distance of 30 cm from the screen was 13.3° in width and 30.7° in height. The face was 20.8° wide and 24.4° high. The distance between the faces depicted in the bilaterally presented videos was 57°.

### 2.3 Procedure

Testing took place in a dedicated room near the neonatal ward. Newborns were seated on the lap of an undergraduate student, unaware of the aims of the study, at a distance of 30 cm from the stimulus presentation monitor (27″ screen size, 1920 × 1080 pixel resolution, 60 Hz). Newborns’ gaze was recorded by a camera placed above the monitor to allow online coding of newborns’ visual behavior. The undergraduate student could check if the newborn position was central with respect to the screen on a small monitor displaying his/her face. Total looking times were measured within a preferential looking paradigm with an infant‐control procedure (Addabbo et al., 2015; Longhi et al., 2015). By simultaneously showing the two hand‐to‐face actions, we can assess whether newborns show a spontaneous visual preference for one of the stimuli, thus demonstrating sensitivity to the difference. Half of the newborns (*N* = 18) were randomly assigned to the mouth‐opening condition and the other half (*N* = 18) to the mouth‐closed condition. Newborns were presented with two trials, in which the thumb‐to‐mouth and thumb‐to‐chin actions were displayed simultaneously and bilaterally on the screen. Each trial began as soon as the newborns looked at a flickering red circle appearing in the center of the monitor. The left/right position of the videos was counterbalanced between the first and the second trial and across participants. Each trial ended when the newborn watched each stimulus at least once and shifted their gaze away for more than 10 s. At the end of the testing session, we asked newborns’ mothers to score on a 5 point Likert scale (from 1 = never, 2 = rarely, 3 = sometimes, 4 = often, 5 = very often) how frequently they have seen their newborn bringing the hand to the mouth. Results showed that hand‐to‐mouth contacts were already observed at birth (*M* = 3.3; *SD* = 1.2), being the scores significantly different from zero (one‐sample *t*‐test vs. 0, *t_(35)_*_ _= 15.95, *p *< 0.001). In particular, 11,1% of the mothers responded “Never,” 13,9% “Seldom,” 27,8% “Sometimes,” 30,6% “Often,” and 16,7% “Very often.” Thus, the majority of the mothers (75,1%) reported that their newborns performed hand‐to‐mouth gestures from “sometimes” to “very often.” Importantly, there was no difference between the scoring of newborns’ experience in the mouth‐opening and closed‐mouth conditions (*t*‐test, *t(34)* = 0.267, *p* = 0.80). The video recordings of eye movements of 50% of the sample were coded offline by an Experimenter, blind to the stimuli shown. The inter‐coder agreement (Pearson correlation) with a second experimenter was 0.97 for total fixation time. The intra‐class correlation coefficient was 0.98.

## 3 RESULTS

**FIGURE 2:**
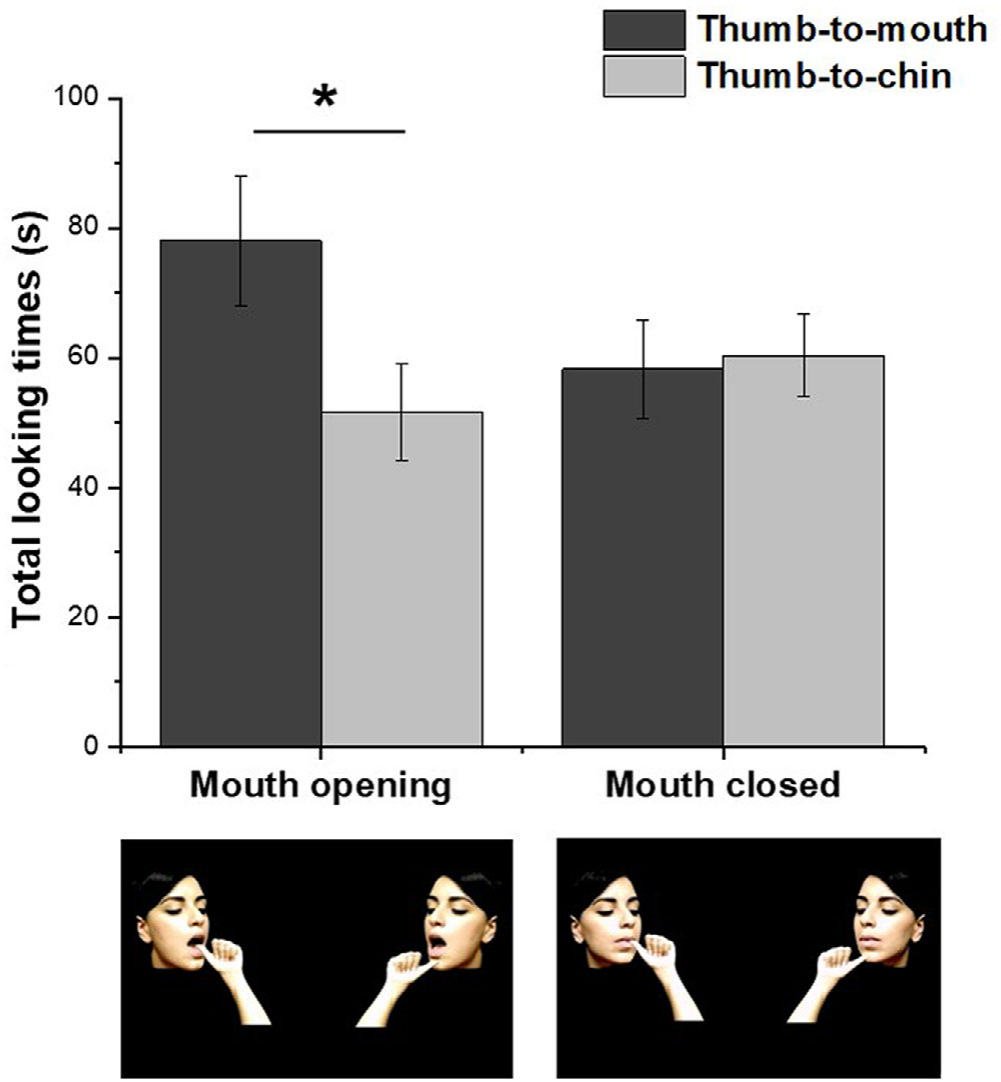
Total looking times towards the thumb‐to‐mouth and thumb‐to‐chin action in the mouth‐opening and mouth‐closed condition. Error bars refer to the standard errors of the mean; *p < 0.05

Given that the data were not normally distributed, as assessed by a Kolmogorov–Smirnov test (*p* < 0.05), total fixation times were log‐transformed to normalize their distribution (Csibra et al., 2016). A repeated‐measures analysis of variance (rmANOVAs) was performed with *Trial* (first vs. second) and *Target of the Action* (mouth vs. chin) as within‐subjects factors and *Condition* (mouth‐opening vs. mouth‐closed) as between‐subjects factors. The analysis showed a significant *Target of the Action x Condition* interaction, *F(1,34) *= 5.16, *p* = 0.029, *η_p_^2^* = 0.132 (Figure 2). All the other main effects and interactions were not significant (all *p*s > 0.151). Planned *t*‐test (two‐tailed) indicated that newborns looked longer towards the thumb‐to‐mouth action (*M* = 77.9 s; *SD* = 43.8) compared to the thumb‐to‐chin action (*M* = 51.6 s; *SD* = 31.4) in the mouth‐opening condition, *t(1,17) *= 2.82, *p* = 0.01, *d* = 0.664, 95% *CI* [0.144, 1.169]. Conversely, no significant difference was found between thumb‐to‐mouth (*M* = 58.3 s; *SD* = 32.5) and thumb‐to‐chin (*M* = 60.5 s; *SD* = 26.9) stimuli in the mouth‐closed condition, *t(1,17) *= 0.77, *p* = 0.45, *d* = 0.182, 95% *CI* [−0.646, 0.286]. Results were further confirmed by examination of the data for individual infants, showing that 14 out of 18 infants in the mouth‐opening condition looked longer at the thumb‐to‐mouth compared to the thumb‐to‐chin action (binomial test, *p* = 0.017). Differently, in the mouth‐closed condition only 8 out of 18 infants looked longer at the thumb‐to‐mouth compared to the thumb‐to‐chin action (binomial test, *p* = 0.17).

We ran a further analysis to explore whether newborns’ looking times towards the thumb‐to‐mouth gesture in the mouth‐opening condition were driven by the last part of the video, in which there were differences in local contrast in the region of the mouth. Newborns’ log‐transformed total looking times were measured in a first‐time window in which the hand moved towards the face and reached the face (from 0 to 2640 ms) and in a second‐time window in which the hand entered the mouth/chin (from 2640 to 3640 ms). An rmANOVA with *Time window* (first vs. last), *Trial* (first vs. second), and *Target of the Action* (mouth vs. chin) as within‐subjects factors revealed a significant main effect of *Time window F(1,17)* = 222.6, *p* = 0.001, *η_p_^2^* = 0.92. Newborns looked longer during the first (*M *= 96.1 s; *SD* = 50.9) compared to the second (*M* = 33.4 s; *SD* = 16.8) time window. Further, there was a significant main effect of *Target of the Action*, *F(1,17) *= 6.64, *p* = 0.02, *η_p_^2^* = 0.28. Thus, in both time windows, newborns showed a similar visual behavior manifesting a preference towards the thumb‐to‐mouth action (*M* = 77.9 s; *SD* = 43.8) compared to the thumb‐to‐chin action (*M* = 51.6 s; *SD* = 31.4). To further support this analysis, we conducted separate rmANOVAs across the two time‐windows. Results revealed a significant main effect of Condition in both the first (*F(1,17) =* 5.55, *p* = 0.03, *η_p_^2^* = 0.25) and the second (*F(1,17)* = 4.96, *p* = 0.04, *η_p_^2^* = 0.23) time window. In both time windows newborns looked longer to the thumb‐to‐mouth action (first time window: *M* = 58.1 s; *SD* = 38.8; second time window: *M* = 19.7 s; *SD* = 8.7) compared to the thumb‐to‐chin action (first time window: *M* = 37.9 s; *SD* = 23.2; Second time window: *M* = 13.7 s; *SD* = 9.2). No other effects reached significance (first time window: all *p*s > 0.32; second time window: all *p*s > 0.74). These findings exclude the possibility that newborns’ attention was biased toward thumb‐to‐mouth gestures only in the last short portion of the video in which the hand entered the mouth/chin and because of a slight difference in local contrast related to a specific portion of the observed action. Rather, our results suggest that newborns are sensitive to the overall target‐relatedness of the observed action.

## 4 DISCUSSION

In the present study, we explored newborns’ early visual sensitivity for face‐related hand actions. Two‐day‐old newborns were presented with thumb‐to‐mouth and thumb‐to‐chin actions in the presence and absence of the mouth opening before hand‐to‐face contacts. Our results show that newborns looked longer towards the thumb‐to‐mouth compared to the thumb‐to‐chin action, only when the mouth opened in anticipation of the hand arrival. Newborns’ visual behavior was not driven by mere perceptual differences in the trajectory and kinematics of the actions nor to a preference to a specific facial region of the face being targeted by the hand. In fact, such preference disappeared in the mouth‐closed condition, in which action kinematics, trajectory, and targets (mouth vs. chin) were kept equal to the mouth‐opening condition.

Thanks to daily hand‐to‐mouth activities, newborns might have learned the contingencies between the opening of the mouth and the hand (or fingers) arrival. This leads to the intriguing suggestion that newborns, in the present study, could have detected the congruency of the observed action with their sensorimotor experience. Since fetal life, newborns open their mouth before bringing their hand, or thumb, in contact with it (Reissland et al., 2014). Hand‐to‐mouth actions remain a prominent behavior at birth. However, differences in the kinematic profile of such movements were found between pre‐ and postnatal life, possibly due to the differences between the intra‐ and extrauterine environment (Zoia et al., 2013). Indeed, at birth, the newborn has to deal with a new environment, richer in stimulations and characterized by an unlimited space and a greater influence of the force of gravity. Throughout the extensive hand‐to‐mouth experience, newborns might have learned to associate the mouth opening with the temporally consequent hand arrival. In the opening‐mouth condition, only actions in which the mouth movement was followed by a thumb‐to‐mouth contact were congruent with newborns’ sensorimotor experience. Thus, newborns’ preference for the thumb‐to‐mouth compared to the thumb‐to‐chin action could be interpreted as newborns’ preference for the action that more closely matched their sensorimotor experience, characterized by the mouth opening followed by hand‐to‐mouth contacts. The opening of the mouth could be considered, then, a cue suggesting the region targeted by the action on the face. Indeed, preference for the thumb‐to‐mouth action, that is, for the familiar action‐outcome association, disappeared when we removed such a critical visual cue. Our results are in line with previous studies showing that sensitiveness to mouth movements is traceable since birth, being newborns able to imitate mouth openings modeled by a live actor (Meltzoff & Moore, 1983). Importantly, such early imitative behavior has been interpreted as the result of newborns' ability to detect the similarity between their own facial movements and those they see performed by adults (the like‐me hypothesis, Meltzoff, 2007). The presence of overlapping representations of ones' own and others' motor acts is also supported by previous evidence that has shown that already at birth, newborns can detect the differences between a hand movement they are able to perform and an impossible hand movement (Longhi et al., 2015). These findings suggest that a rudimentary mechanism that allows newborns to match their own and others' movements might already be in place at birth (Longhi et al., 2015).

The hand movement directed towards the opened mouth might also represent a very salient visual event for the newborn, irrespectively of its congruency/incongruency with their sensorimotor experience. Prenatal hand‐into‐mouth gestures already possess some characteristics similar to behaviors that emerge later in development. Indeed, early hand‐into‐mouth coordinated actions are considered at the roots of feeding behaviors (Lew & Butterworth, 1995; Radzyminski, 2005). Others suggest that fetal self‐exploration and thumb‐sucking could represent primitive forms of soothing behaviors and might be functional to regulate fetal and newborns’ levels of arousal (Feldman & Brody, 1978). Hand‐into‐mouth actions are indeed rich in sensory feedback, being the mouth a very sensitive area of the face (Fagard et al., 2018). Pre‐ and postnatal motor practice during hand‐to‐face actions might allow the newborn to acquire extensive knowledge about the sensory consequences generated by bringing the hand or thumb in the mouth (Fagard et al., 2018). This, in turn, could lead to the emergence of early visual preferences for actions directed towards the opened mouth, compared to other less sensitive regions of the face. However, it is also important to note that newborns’ frequency and duration of breastfeeding experience could have had a role in driving newborns’ attention toward thumb‐to‐open mouth gestures, and future studies should take into account such feeding experience.

Lastly, it could be hypothesized that newborns already possess, since birth, an abstract ability to attribute goals to the observed events (Gergely & Csibra, 2003). Newborns could have recognized the hand movement towards the opened mouth as a goal‐directed action, and their preference might have been driven by a crucial and clearly visible cue, such as the mouth opening. This interpretation implies that, since birth, newborns might also be able to attribute goals to actions that are not part of their experience. Thus, further studies might investigate whether newborns are capable to discriminate similar actions performed by abstract, non‐human agents not resembling the human body. However, it is important to note that a previous finding reported that newborns process differently touching gestures involving body parts (hand‐to‐hand touches) or objects (object‐to‐hand touches). Indeed, newborns were able to discriminate between touching and non‐touching gestures only when presented with body‐related information (Addabbo et al., 2015). We would, therefore, expect the findings of the present study to be limited to body‐related stimuli only.

Overall, we suggest that prenatal experience with hand‐to‐face actions might be responsible for the development of highly organized behaviors at birth and also for the emergence of early visual sensitivities to observed face‐related actions. When practicing hand‐to‐mouth actions, newborns might learn associations between the mouth opening, the hand moving towards the mouth, and the sensory feedback generated by hand‐to‐mouth contact. The sensory effect of such action might be, in turn, associated with specific changes in the newborns’ behavioral states. Such acquired sensorimotor and somatosensory knowledge might serve to build the first rudimentary action‐outcome associations at birth, which, later in life, will develop in sophisticated online predictions and representations of others’ intentions. In fact, it has been shown that the ability to anticipate through motor resonance object‐to‐mouth actions gradually develops from 3 to 9 months of age (Natale et al., 2014; Turati et al., 2013), showing that experience has a role in infants’ action‐prediction abilities. A related study showed that infants visually anticipate feeding actions earlier than other manual actions with which they had no or limited experience (i.e., combing the hair) (Gredebäck & Melinder, 2010; Kochukhova & Gredebäck, 2010). Noteworthy, infants use different cues to anticipate the goal of an action. For example, they can use object information (Reid et al., 2009) or hand shape and action kinematics (Ambrosini et al., 2013; Filippi & Woodward, 2016; Stapel et al., 2015) to predict the outcome of an action. Here, we hypothesized that the mouth opening could be considered a crucial visual cue on which newborns could rely to associate an action to its specific target. Even if, at birth, newborns might not be able to represent the goal of an observed action, they might already possess the ability to visually associate sensorimotor events experienced as contingent during their daily hand‐to‐mouth experiences. However, such interpretation needs to be confirmed by further studies. For example, future studies might explore more in‐depth newborns’ spontaneous preference for hand‐to‐mouth actions manipulating the temporal contingency between the mouth opening and hand arrival.

To sum up, our finding suggests that already at birth, newborns are particularly attuned to actions directed towards the mouth when preceded by the mouth opening. Our results show that newborns can discriminate between body‐related actions directed towards different regions of the face, and such early spontaneous preference might arise from primitive sensorimotor and somatosensory associations experienced on their own body and transferred to the visual modality. Our finding represents the first step towards a better understanding of newborns' early sensitivity to hand‐to‐mouth actions. However, future studies are needed to confirm our interpretations and to have a full comprehension of the mechanism that could have driven newborns' visual preference towards the thumb‐to‐mouth opened action.

## CONFLICT OF INTEREST

We certify that there are no affiliations with or involvement in any organization or entity with a direct financial interest in the subject matter or materials discussed in the manuscript (e.g., employment, consultancies, stock ownership, honoraria, and/or expert testimony).

## Supporting information

Supporting information.

Supporting information.

## Data Availability

The data that support the findings of this study are available from the corresponding author.
